# Prognostic and clinicopathological significance of NRF2 expression in non-small cell lung cancer: A meta-analysis

**DOI:** 10.1371/journal.pone.0241241

**Published:** 2020-11-13

**Authors:** Qingsong Wang, Liang Xu, Gang Wang, Lei Chen, Changping Li, Xiangli Jiang, Hai Gao, Bing Yang, Weiping Tian

**Affiliations:** 1 Guangdong-Hongkong-Macau Institute of CNS Regeneration, Jinan University, Guangzhou, China; 2 Department of Obstetrics and Gynecology, Tianjin Medical University General Hospital, Tianjin, China; 3 Department of Otolaryngology, The Second Hospital of Tianjin Medical University, Tianjin, China; 4 Department of Health Statistics, College of Public Health, Tianjin Medical University, Tianjin, China; 5 Department of Thoracic Medical Oncology, Tianjin Medical University Cancer Institute and Hospital, Tianjin, China; 6 Institutes of Biomedical Sciences, Fudan University, Shanghai, China; 7 Department of Cell Biology, School of Basic Medical Sciences, Tianjin Medical University, Tianjin, China; 8 Tianjin Research Center of Basic Medical Sciences, School of Basic Medical Sciences, Tianjin Medical University, Tianjin, China; Qatar University College of Medicine, QATAR

## Abstract

Nuclear factor erythroid 2-related factor 2 (NRF2) functions as a transcription factor and regulates a wide array of antioxidant and stress-responsive genes. NRF2 has been widely implicated in different types of cancers, but only limited studies concerning the relationship between NRF2 expression and tumour invasion or prognosis in lung cancer. Therefore, we conducted a meta-analysis to determine the prognostic value of NRF2 in patients with non-small cell lung cancer (NSCLC). The relationship between NRF2 expression in NSCLC patients and clinicopathological features was also investigated. Overall survival (OS) and treatment response rate were evaluated using STATA software. Twenty eligible articles with 2530 lung cancer patients were included in this meta-analysis. The results revealed that high expression level of NRF2 was associated with pathologic distant metastasis (odds ratio (OR) = 2.64, 95% confidence interval (CI) 1.62–4.31; P < 0.001), lymph node metastasis (OR = 2.14, 95% CI: 1.53–3.00; P < 0.001), and tumour node metastasis (TNM) stage (OR = 1.95, 95% CI: 1.52–2.49, P < 0.001). High NRF2 expression was associated with low treatment response rate in platinum-based chemotherapy (HR = 0.11, 95% CI 0.02–0.51; P = 0.005). High expression level of NRF2 is predictive for poor overall survival rate (HR = 1.86, 95% CI 1.44–2.41, P < 0.001) and poor progression-free survival (PFS) (HR = 2.27, 95% CI 1.26–4.09, P = 0.006). Compared to patients with a low level of NRF2 expression, patients with high NRF2 expression levels were associated with worse OS and PFS when given the chemotherapy or EGFR-TKI. Together, our meta-analysis results suggest that NRF2 can act as a potential indicator of NSCLC tumour aggressiveness and help the prognosis and design of a better treatment strategy for NSCLC patients.

## Introduction

Nuclear factor erythroid 2 like 2 (NRF2), also known as nuclear factor erythroid 2-related factor 2 (NFE2L2), is a transcription factor encoded by the *NRF2* gene in humans [1]. NRF2 regulates the transcription of a wide array of genes, including those coding for antioxidant proteins involved in the detoxification of xenobiotics and resistance to oxidative stress [2]. For example, both heme oxygenase-1 (HO-1) and NAD(P)H quinone oxidoreductase 1 (NQO1) are regulated by NRF2. The cytoplasmic NRF2 protein is maintained at a very low level through its selective negative regulator, Kelch-like ECH-associated protein 1 (KEAP1). KEAP1 can sequester NRF2 in the cytoplasm and lead to ubiquitination of CUL3 E3 ligase and subsequent degradation by the proteasome [3,4]. Under oxidative stress or in the presence of NRF2-activated compounds, E3 activity is downregulated and NRF2 is stabilized, thereby increasing the amount of NRF2 protein relative to KEAP1 [5,6]. The free NRF2 translocates to the nucleus, then activates the expression of its downstream antioxidant genes [7,8].

NRF2 signalling is crucial for the initiation and progression of lung cancer, as shown by gene knockout mouse model and clinical studies [9]. An enhanced NRF2 signal activity appears to be correlated with a worse treatment outcome according to the clinical observations [10]. In cancer cells, NRF2 signalling can be activated by endogenous or exogenous stress, accompanied by activation of various cytoprotective genes [11]. Furthermore, crosstalk has been reported between NRF2 and oncogenic signaling pathways such as phosphatidylinositol 3-kinase (PI3K) [12], Kirsten retrovirus-associated DNA sequence (K-RAS) [9], and Notch [13].

Many studies have evaluated whether the positive expression of NRF2 may be a prognostic factor for survival rate among patients with lung cancer. However, the clinical evidence for the relationship between NRF2 expression and tumour invasiveness, prognosis and treatment response rate in NSCLC is not well understood. In this study, a meta-analysis of published data was performed to systematically investigate whether NRF2 expression can be an applicable marker to assist with the prognosis of patients with NSCLC.

## Materials and methods

### Literature search strategy

This meta-analysis was conducted in accordance with the PRISMA guidelines [14]. The Chinese databases of China National Knowledge Infrastructure (CNKI) as well as English databases of Pubmed, Embase, EBSCO and the web of science were retrieved from inception to May 25, 2020, using combinations of the following keywords: (“NRF2” OR “NFE2L2” OR “nuclear factor erythroid-2-related factor 2”) AND (“Non-Small Cell Lung Carcinoma” OR “lung cancer” OR “lung squamous cell carcinomas” OR “Lung Adenocarcinomas”). Pubmed search terms are shown in S1 Table.

### Inclusion criteria

**Fig 1:**
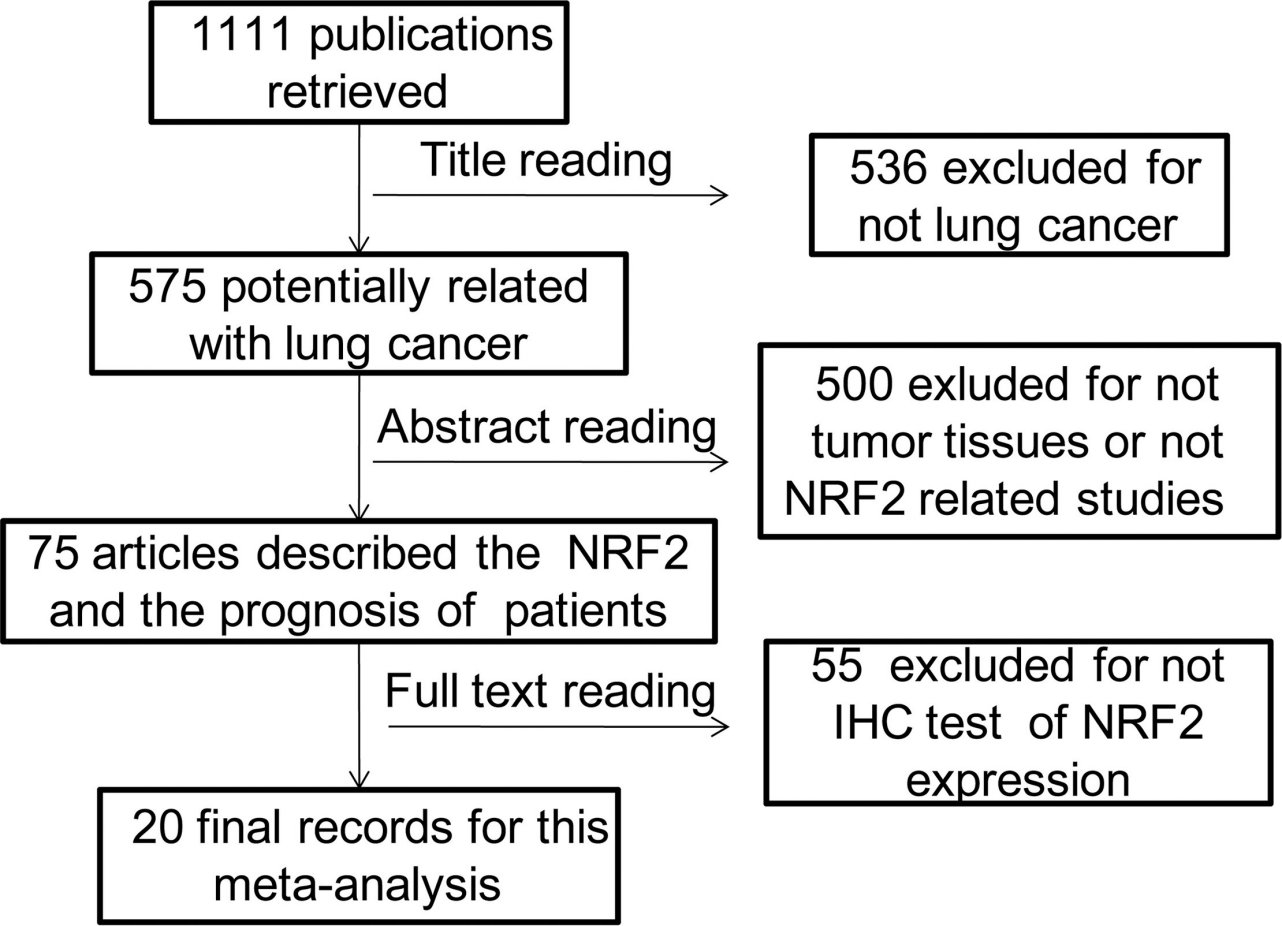
Flow diagram of study selection process.

Studies eligible for inclusion in this meta-analysis met the following criteria: (1) measure NRF2 expression in the primary lung cancer with IHC (immunohistochemistry); (2) provide enough clinicopathological parameters or hazard ratio (HR) and 95% confidence interval (CI) between NRF2 expression and OS; (3) the minimum sample size was 30; (4) data specifically focus on NSCLC was extracted. Following the search, 20 articles were selected for our analysis (Fig 1). All the articles are retrospective study.

### Data extraction

Data extraction and information on study design, outcomes were performed by two independent observers (Qingsong Wang and Liang Xu) and disagreements were settled by discussion and consensus with a third author (Bing Yang).

As for each study, the following information was extracted: the name of the first author, year of publication, country, and number of cases, gender, expression location, cut-off value of NRF2, detection method, positive percentage, treatment, clinicopathological features, and the related survival data. Calculation method introduced by Tierney et al [15] and Parmar et al [16] was applied to extract HR with 95% CI where HR was not reported. Quality evaluation was based on the Newcastle-Ottawa quality assessment scale (NOS). The studies with NOS scores ranging from 6 to 9 were deemed as high quality. The summary of included studies can be found in Table 1.

**Table 1:** The basic information and data of included studies.

No.of Studies	First Author	Year	Country	Sample Size	Gender(M/F)	Location	Cut-off value	Detection method	NRF2 Positive Percentage	Treatment	NOS Score
1	Luisa M. Solis [11]	2010	U.S	304	157/147	Nuclear	score >0	IHC(Santa Cruz)	26.0%	C	9
2	Haihong Yang [10]	2011	China	60	40/20	Cytoplasmic	The cells stained ≥50%	IHC(Beijing Biosynthesis)	56.7%	C	9
3	Daisuke Inoue [25]	2012	Japan	109	78/31	Nuclear	The cells stained >10%	IHC(Santa Cruz)	33.9%	N.A	9
4	Heta Merikallio [17]	2012	Finland	289	N.A	Cytoplasmic	The cells stained≥50%	IHC(Santa Cruz)	N.A	N.A	8
5	Ming-Hsien Chien [25]	2015	Taiwan	167	64/103	Nuclear	The cells stained >20%	IHC(Cell Signaling)	52.1%	N.A	9
6	Xiang Zhu [19] a	2014	China	31	10/21	Nuclear	score >0	IHC(Abcam)	77.4%	T	9
6`	Xiang Zhu [19] b	2014	China	31	10/21	Cytoplasmic	score >2	IHC(Abcam)	38.7%	T	9
7	Joo-Heon Kim [20]	2007	U.S	89	44/45	Cytoplasmic	The cells stained≥25%	IHC(Abcam)	61.8%	N.A	8
8	Tinghua Hu [26]	2014	China	66	50/16	Nuclear	IRS ≥ 4	IHC(Abcam)	63.6%	N.A	7
9	Baoshan Cao [27]	2012	China	50	29/21	Nuclear	score >0	IHC(Beijing Biosynthesis)	34.0%	C	9
10	Jing Wang [28]	2017	China	80	42/38	Nuclear	IRS ≥ 4	IHC(Beijing Biosynthesis)	66.2%	N.A	9
11	Shou Yu [29]	2018	China	116	60/56	Nuclear	IRS ≥ 4	IHC(Abcam)	62.1%	N.A	8
12	Qingkay Li [30]	2011	US	55	N.A	Cytolasmic	N.A	IHC(Santa Cruz)	85.5%	N.A	7
13	Ying-Hui Tong [31]	2017	China	215	170/45	nuclear	The cells stained≥10%	IHC(Santa Cruz)	68.4%	C	8
14	Jueshi Liu [32]	2018	China	72	46/26	nuclear	≥2 score	IHC(Beijing Biosynthesis)	41.7%	N.A	7
15	Yu Xiao [33]	2018	China	104	47/57	nuclear	score >0	IHC(Abcam)	71.2%	T	7
16	Xueying Zhu [34]	2018	China	92		nuclear	score >0	IHC(Abcam)	73.9%	N.A	7
17	Manqing Liu [35]	2018	China	130	89/41	cytoplasmic	The cells stained≥10%	-	64.6%	C	9
18	Ying E [36]	2019	China	72	41/31	cytoplasmic	score ≥ 4	IHC(Abcam)	62.5%	C	9
19	Hongyan Wang [37]	2019	China	95	43/52	nuclear	IRS ≥ 5	IHC(Santa Cruz)	60.0%	N.A	8
20	Ming-Jen Chen [24]a	2020	Taiwan	167	113/54	cytoplasmic-nuclear	N.A	IHC(GeneTex)	19.0%	C	8
20`	Ming-Jen Chen [24] b	2020	Taiwan	167	113/54	cytoplasmic	N.A	IHC(GeneTex)	53.0%	C	8

Abbreviations: C: chemotherapy; T: EGFR-TKI (Epidermal growth factor receptor tyrosine kinase inhibitor)., N.A: not available; IRS = SI (staining intensity) ×PP (percentage of positive cells).

### Statistical analysis

All the statistical data were analyzed using STATA software (Version 12.0; Stata Corporation). Pooled odds ratios (ORs) with 95% CIs were calculated to evaluate the association between positive NRF2 expression and clinicopathological features (gender (male *vs*. female), smoking (current and former *vs*. never), histopathology (SCC *vs*. AC), differentiation type (poor/undifferentiated *vs*. well/moderate), TNM stage (TNM, III~IV *vs*. I~II), TNM stage (IV *vs*. III), lymph node metastasis (Yes *vs*. No), treatment response rate (CR/PR *vs*. SD/PD). HRs with a 95% CI in OS and PFS were calculated to evaluate the relationships between positive NRF2 expression and the prognosis of lung cancer patients. The heterogeneity among the enrolled studies was evaluated by I^2^ and Q statistic. The P value> 0.10 and I^2^ <40% were taken as a lack of heterogeneity. A logistic random-effect model was utilized for the studies with a significant heterogeneity (P≤0.10, I^2^≥40%). The sensitivity analysis was carried out owing to the relatively significant heterogeneity among the studies. Moreover, subgroup analyses were used to investigate potential sources of heterogeneity. Publication bias was evaluated by Begg test. A value of *P*<0.05 was considered statistically significant.

## Results

### Literature search

After the initial search algorithm, there were 507 publications from Web of science, 95 more articles were added from the Pubmed, seven publications were added from EBSCO and 40 were added from EMBASE with duplicates removed. Among them, there were 28 meeting abstracts, so 621 from English database. There were 698 publications from CNKIs. The total of 1111 potentially relevant studies were selected from the English database and CNKI databases using criteria as defined in the methods. Five hundred thirty-six articles were excluded as non-original studies (review) and non-lung-cancer studies. The remaining 575 articles were further assessed by screening the abstracts, 500 of which were excluded because they were concerned with non-human tumour tissue specimens. Seventy-five studies were included for full-text assessment. A further 55 studies were excluded for lacking the immunological histological chemistry (IHC) test of NRF2 expression. Finally, a total of 20 eligible articles with 2530 NSCLC patients were included in this meta-analysis [10,11,17–23]. The schematic process of literature selection is shown in Fig 1.

### The primary characteristics of studies

Twenty eligible studies published between 2007 and 2020 used IHC methodology to evaluate the expression level of NRF2 in human NSCLC tissues. The studies were conducted in five countries or regions. Fourteen studies reported the prognostic value of NRF2 status for survival in patients with NSCLC. The location of NRF2 expression within the cell was described differently in the various studies. In the present study, these articles are described as either ‘nuclear location’ or ‘cytosolic location’ depending on the results presented in the source articles. NO.6 Xiang Zhu’s study contains two sets of data including both nuclear and cytosolic locations of NRF2 expression from the same group of patients, so we treated it as two independent studies [19]. NO.20 Ming-Jen Chen’s study contains two sets of data including C+/N+ (NRF2 cytoplasmic/ nuclear both positive immunostaining) and C+/N- (Nrf2 cytoplasmic positive/ nuclear negative immunostaining) two different cytosolic locations of NRF2 expression from the same group of patients, so we also treated it as two independent studies [24]. The sample size ranged from 31 to 304, and the percentage of positive NRF2 expression ranged from 19% to 77.4%. The main characteristics of the twenty included studies were summarized in Table 1. The clinical and pathological parameters of all included studies were showed in Table 2.

**Table 2:** The summarized data of clinical and pathological parameters from all included studies in the meta-analysis.

				Gender		Smoking history		TNM Stage		Metastasis		Lymph node metastasis		Cancer type(nuclear positive)		Histological differentiation		Treatment		Response rate		OS		PFS	
No.of Studies	First Author	Year	NRF2 expression	male	Female	Never	Current/former	I + II	III+ IV	M0	M1	Yes	No	Squamous cell carcinomas	Adenocarcinomas	Well and moderately	Poorly/undifferentiated	Yes	No	CR and PR	SD and PD	HR estimate	95% CI	HR estimate	95% CI
1	Luisa M. Solis	2010	High	157	147	50	253	-	-	-	-	-	-	43	34	-	-	34	20	-	-	1.747	1.12–2.726	2.31	1.53–3.47
			Low	-	-	-	-	-	-	-	-	-	-	79	154	-	-	55	39	-	-	-	-	-	-
2	Haihong Yang	2011	High	26	8	12	22	-	-	-	-	-	-	5	29	8	17	24	10	4	13	0.3	0.138–0.652	0.174	0.062–0.448
			Low	14	12	13	13	-	-	-	-	-	-	6	20	8	9	19	7	18	8	-	-	-	-
3	Daisuke Inoue	2012	High	31	6	-	-	-	-	-	-	14	23	11	25	27	10	-	-	-	-	5	2.4–10.6	-	-
			Low	47	25	-	-	-	-	-	-	21	51	20	47	49	23	-	-	-	-	-	-	-	-
4	Heta Merikallio	2012	High	-	-	-	-	-	-	-	-	-	-	71	57	-	-	-	-	-	-	1.49*	1.23–1.79	-	-
			Low	-	-	-	-	-	-	-	-	-	-	36	29	-	-	-	-	-	-	-	-	-	-
5	Ming-Hsien Chien	2015	High	32	55	-	-	33	54	62	25	55	32	-	-	-	-	-	-	-	-	-	-	-	-
			Low	32	48	-	-	47	33	64	16	37	43	-	-	-	-	-	-	-	-	-	-	-	-
6	Xiang Zhu a	2014	High	6	18	16	8	-	-	-	-	-	-	-	-	19	5	24	0	13	11	1.352	0.487–3.752	-	-
			Low	4	3	4	3	-	-	-	-	-	-	-	-	7	0	7	0	6	1	-	-	-	-
6`	Xiang Zhu b	2014	High	3	9	8	4	-	-	-	-	-	-	-	-	9	3	12	0	0	12	5.449	1.065–27.873	5.944	1.912–18.483
			Low	7	12	12	7	-	-	-	-	-	-	-	-	17	2	19	0	19	0	-	-	-	-
7	Joo-Heon Kim	2007	High	23	32	14	41	-	-	-	-	-	-	16	33	30	24	-	-	-	-	-	-	-	-
			Low	21	13	3	31	-	-	-	-	-	-	15	15	12	20	-	-	-	-	-	-	-	-
8	Tinghua Hu	2014	High	35	7	21	21	18	24	28	14	33	9	22	20	15	27	-	-	-	-	-	-	-	-
			Low	15	9	13	11	17	7	22	2	13	11	14	10	11	13	-	-	-	-	-	-	-	-
9	Baoshan Cao	2012	High	9	8	6	11	-	-	3	14	-	-	8	9	17	0	17	0	3	14	1.791	0.933–3.438	2.067	0.649–6.583
			Low	20	13	13	20	-	-	17	16	-	-	15	18	31	2	33	0	14	19	-	-	-	-
10	Jing Wang	2017	High	27	27	-	-	19	35	-	-	36	18	26	28	-	-	-	-	-	-	2.078	1.186–3.641	2.623	1.229–5.599
			Low	15	11	-	-	16	10	-	-	14	12	10	16	-	-	-	-	-	-	-	-	-	-
11	Shou Yu	2018	High	33	39	42	30	30	42	45	27	-	-	48	24	32	40	-	-	-	-	3.734	1.466–9.508	0.8	0.63–1.01
			Low	27	17	27	17	29	15	37	7	-	-	34	10	12	32	-	-	-	-	-	-	-	-
12	Qingkay Li	2011	High	-	-	-	46	16	8	-	-	-	-	-	-	-	-	-	-	-	-	-	-	-	-
			Low	-	-	-	3	27	4	-	-	-	-	-	-	-	-	-	-	-	-	-	-	-	-
13	Ying-Hui Tong	2017	High	116	31	34	98	84	63	-	-	96	51	77	65	77	60	-	-	-	-	1.55	1.2–2.01	-	-
			Low	54	14	13	44	44	24	-	-	28	40	35	32	24	37	-	-	-	-	-	-	-	-
14	Jueshi Liu	2018	High	19	11	-	-	24	6	-	-	9	21	17	13	20	10	-	-	-	-	-	-	-	-
			Low	27	25	-	-	20	22	-	-	26	16	23	19	8	34	-	-	-	-	-	-	-	-
15	Yu Xiao	2018	High	38	36	45	29	23	51	-	-	-	-	-	-	60	12	-	-	-	-	7.505	1.656–34.007	8.487	2.234–32.239
			Low	9	21	15	15	7	23	-	-	-	-	-	-	22	6	-	-	-	-	-	-	-	-
16	Yingxue Zhu	2018	High	-	-	-	-	31	37	-	-	-	-	39	29	-	-	-	-	-	-	-	-	-	-
			Low	-	-	-	-	18	6	-	-	-	-	14	10	-	-	-	-	-	-	-	-	-	-
17	Manqing Liu	2018	High	58	26	47	37	-	92	-	-	-	-	29	39	-	-	-	-	-	-	6.296	1.992–19.899	-	-
			Low	31	15	32	14	-	38	-	-	-	-	19	17	-	-	-	-	-	-	-	-	-	-
18	Ying E	2019	High	26	19	17	28	26	19	-	-	-	-	26	19	15	30	-	-	-	-	2.16	0.65–7.18	-	-
			Low	15	12	9	18	24	3	-	-	-	-	17	10	8	19	-	-	-	-	-	-	-	-
19	Hongyan Wang	2019	High	22	35	-	-	-	-	-	-	-	-	-	-	17	40	-	-	-	-	-	-	-	-
			Low	21	17	-	-	-	-	-	-	-	-	-	-	12	26	-	-	-	-	-	-	-	-
20	Ming-Jen Chen a	2020	High	15	17	23	9	20	12	-	-	-	-	14	18	-	-	-	-	-	-	1.638	1.059–2.535	1.676	1.074–2.614
			Low	98	37	68	69	102	33	-	-	-	-	54	81	-	-	-	-	-	-	-	-	-	-
20'	Ming-Jen Chen b	2020	High	54	34	55	33	64	24	-	-	-	-	35	53	-	-	-	-	-	-	1.568	1.046–2.349	1.609	0.874–1.533
			Low	59	20	36	45	58	21	-	-	-	-	33	46	-	-	-	-	-	-	-	-	-	-

Abbreviations: SCC: squamous cell carcinomas, AC: adenocarcinomas, OS: overall survival, PFS: progression-free survival, HR: hazard ratio, OR: odds ratio, RR: relative risk, CI: confidence interval, EGFR-TKI: Epidermal growth factor receptor tyrosine kinase inhibitor, CR: complete response, PR: partial response, PD: progression of disease, SD: stable disease.

### Association between NRF2 and clinicopathological features in NSCLC

**Fig 2:**
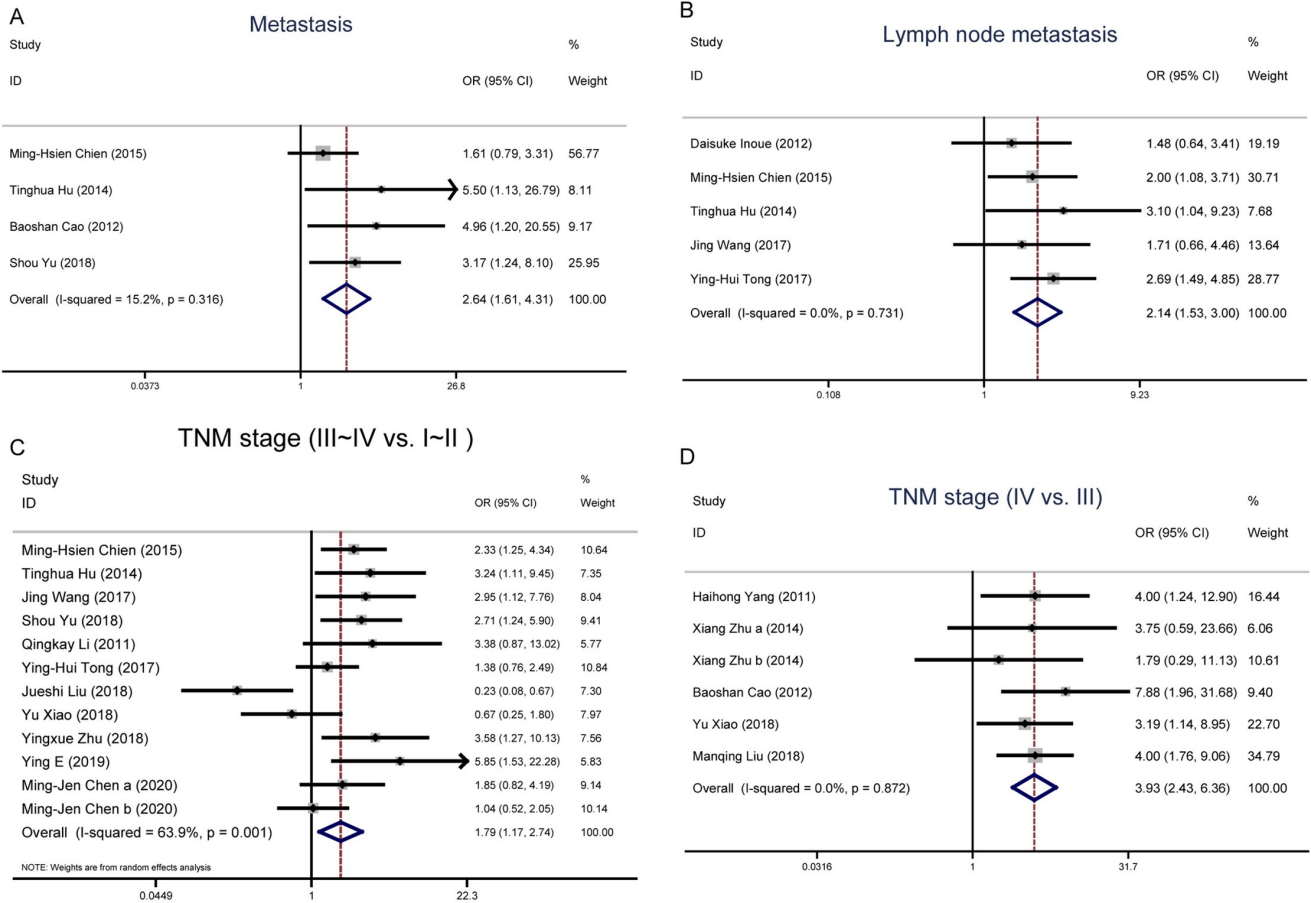
Forest plot of the NRF2 expression level and clinicopathological features. A. Forest plot of studies evaluating the relationship between NRF2 expression and distant pathological metastasis. B. Forest plot of studies evaluating the relationship between NRF2 expression and lymph node metastasis. C. Forest plot of studies evaluating the relationship between NRF2 expression and pathological tumour-node-metastasis (TNM, III~IV *vs*. I~II). D. Forest plot of studies evaluating the relationship between NRF2 expression and pathological tumour-node-metastasis (TNM, IV *vs*. III).

To examine the clinical value of NRF2, we investigated the associations between NRF2 and a number of clinicopathological parameters [18,21,23]. The logistic fixed-effects model was employed because of restricted heterogeneity among the studies (I^2^ = 15.2%, P = 0.316). As seen in Table 3 and Fig 2A pooled odds ratio (OR) values from the four eligible studies showed that upregulated NRF2 was associated with distant metastasis (OR = 2.64, 95% CI 1.62–4.31; P<0.001).

**Table 3:** Main results and publication bias for meta-analysis between NRF2 and clinicopathological features, overall survival (OS) and PFS (progression-free survival).

Correlation between NRF2 and clinicopathological features / OS/PFS	No. of studies	Overall OR/HR (95%CI)	*z*,*P*_OR/HR_	Heterogeneity test (*I*^2^, *P*_bias_)	Publication bias (Egger’s test)
					(*t*, *P*_publication bias_)
Metastasis (M1/M0)	5, 8, 9, 11	2.64 (1.62,4.31)	3.87, < 0.001	15.2%, 0.316	4.04, 0.056
Lymph node metastasis (Yes *vs*. No)	3, 5, 8, 10, 13	2.14 (1.53, 3.00)	4.46 < 0.001	0.0%, 0.731	-0.33, 0.764
TNM stage (III~IV *vs*. I~II)	5, 8, 10, 11, 12, 13, 14, 15, 16, 18, 20 20'	1.79 (1.17, 2.74)	2.68, 0.007	63.9%, 0.001	0.53, 0.607
TNM stage (IV *vs*. III)	2, 6, 6', 9, 15, 17	3.93 (2.43, 6.36)	5.58, < 0.001	0.0%, 0.872	-0.29, 0.787
Treatment response rate (CR/PR *vs*. SD/PD)	2, 6, 6', 9	0.11 (0.02, 0.51)	2.84, 0.005	58.0%, 0.067	-2.06, 0.175
OS	1, 2, 3, 4, 5, 6, 6', 9, 10, 11, 13, 15, 17, 18, 20, 20'	1.86 (1.44, 2.41)	4.73, < 0.001	67.9%, < 0.001	1.65, 0.122
PFS	1, 2, 6', 9, 10, 11, 15, 20	2.27 (1.26, 4.09)	2.74, 0.006	86.2%, < 0.001	3.53, 0.017
Gender (male *vs*. female)	2, 4, 5, 6, 6', 7, 8, 9, 10, 11, 13, 14, 15, 17, 18, 19, 20, 20'	0.90 (0.66, 1.23)	0.65, 0.515	53.6%, 0.004	0.78, 0.448
Smoking (current and former *vs*. never)	2, 6, 6', 7, 8, 9, 11, 13, 15, 17, 18, 20, 20'	1.23 (0.96, 1.58)	1.68, 0.094	26.4%, 0.178	-1.52, 0.157
Histopathology (SCC *vs*. AC)	1, 2, 3, 4, 7, 8, 9, 10, 11, 13, 14, 16, 17, 18, 20, 20'	1.05(0.86, 1.27)	0.44, 0.657	16.6%, 0.264	-2.46, 0.028
Differentiation type (poor/undifferentiated *vs*. well/moderate)	2, 3, 6, 6', 7, 8, 9, 11, 13, 14,15, 18, 19	1.48 (0.95, 2.30)	1.73, 0.083	47.3%, 0.035	-1.23, 0.247

Abbreviations: SCC: squamous cell carcinomas, AC: adenocarcinomas, OS: overall survival, PFS: progression-free survival, HR: hazard ratio, OR: odds ratio, CI: confidence interval, EGFR-TKI: Epidermal growth factor receptor tyrosine kinase inhibitor, CR: complete response, PR: partial response, PD: progression of disease, SD: stable disease.

The patients with lymph node metastasis based on different levels of NRF2 expression was reported in 6 studies. The logistic random-effects model was applied because of significant heterogeneity in the studies (I^2^ = 71.0%, P = 0.004). The analysis showed a pooled OR = 1.55 (95%CI: 0.84–2.87, P = 0.164). Owing to the relatively severe heterogeneity among the studies on lymph node metastasis, the sensitivity analysis and published bias were carried out, in which the Jueshi Liu’s study in 2018 was the cause of statistical heterogeneity (S2 Fig). When this study was removed, the heterogeneity disappeared in the remaining studies (P = 0.731, I^2^ = 0%). The analysis of these left studies indicated a statistically obvious association between the high NRF2 expression and the lymph node metastasis. The combined OR estimates were 2.14 (95% CI: 1.53–3.00; P < 0.001). This analysis suggests that heterogeneity among different studies should be treated with extra caution when interpreting it (Fig 2B).

The patients with TNM stage (TNM, III~IV *vs*. I~II) based on different levels of NRF2 expression was reported in 11 studies. The logistic random-effects model was applied because of significant heterogeneity in the studies (I^2^ = 63.9%, P = 0.001). The analysis showed a pooled OR = 1.79 (95%CI: 1.17–2.74, P = 0.007), as shown in Fig 2C. The sensitivity analysis was carried out owing to the relatively large heterogeneity among the studies on the TNM stage. The pooled OR estimates were consistent without distinct fluctuation. The results demonstrated that high expression level of NRF2 was related to the advanced TNM stage. Then, we further analyzed the NRF2 expression in advanced TNM stage (TNM, IV *vs*. III). There are five studies with TNM IV and III patients. The logistic fixed-effects model was employed because of restricted heterogeneity among the studies (I^2^ = 0.0%, P = 0.872). The meta-analysis demonstrated a combined OR = 3.93 (95%CI: 2.43–6.36, P<0.001), as shown in Fig 2D. The results demonstrate that the advanced TNM stage is distinctly related to the high NRF2 expression level.

On the other hand, the relationships between NRF2 expression level and gender (OR = 0.90, 95%CI 0.66–1.23; P = 0.515), smoking (OR = 1.23, 95%CI 0.96–1.58; P = 0.094), histopathology (OR = 1.05, 95%CI 0.86–1.27; P = 0.657), and differentiation type (OR = 1.48, 95%CI 0.95–2.30; P = 0.083) were not significant (Table 3, S1 Fig).

### Association between NRF2 expression level and treatment response rate in NSCLC

Four studies included data relating to the response to treatment. These studies were assessed for the association between NRF2 and treatment response rate in NSCLC. Two studies elucidated NRF2 expression and outcome in patients treated with platinum-based chemotherapy. The other two studies determined that NRF2 was a good biomarker for predicting response to EGFR-TKI (Epidermal growth factor receptor tyrosine kinase inhibitor) in patients with EGFR gene mutations. All the patients included are in stage III or stage IV NSCLC, and 17 patients had undergone surgery in one of the chemotherapy studies. Our results indicate that the high NRF2 expression level was associated with treatment response rate (OR = 0.11, 95%CI 0.02–0.51; P = 0.005). However, heterogeneity was found to be relatively large (*I*^*2*^ = 58.0%, p^*bias*^ = 0.067) (Table 3).

**Fig 3:**
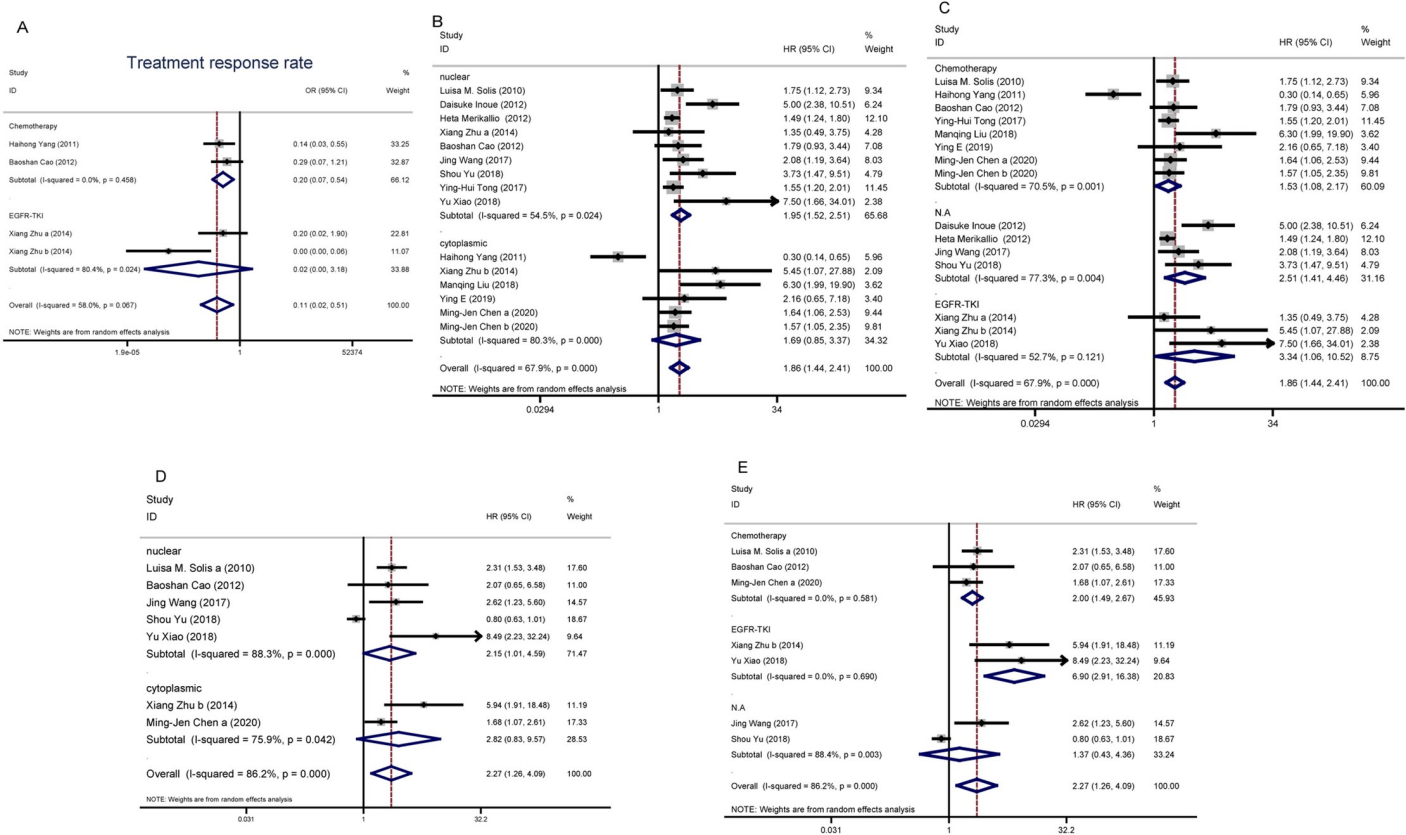
The association between NRF2 high expression and treatment response rate, overall survival and progression-free survival in NSCLC. A. Forest plot for subgroup study about treatment response rate. B. C. Forest plot for the subgroup study about relationship between NRF2 expression and overall survival (OS). D.E. Forest plot of subgroup study about evaluating the relationship between NRF2 expression and progression free survival (PFS).

Subgroup analysis was performed on treatment method to explore the potential sources of heterogeneity. As seen in Table 4 and Fig 3A, the results showed that there was no relationship between NRF2 expression and treatment response rate (OR = 0.02, 95%CI 0.01–3.18; P = 0.13) in the EGFR-TKI treated group. In the chemotherapy-treated group, upregulated NRF2 was associated with a low treatment response rate (OR = 0.20, 95%CI 0.07–0.54; P < 0.01). Heterogeneity was not a major factor in the chemotherapy treated group (*I*^*2*^ = 0.0%, *P*^*bias*^ = 0.458). Taken together, the heterogeneity of treatment response rate was mainly caused by the different treatment methods. The high NRF2 expression level was associated with a low treatment response rate in platinum-based chemotherapy.

**Table 4:** Subgroup analysis of treatment response rate, overall survival and progression free survival.

Subgroups	Studies	OR/HR(95%CI)	z	*P*_OR/HR_	*I*^2^	*P*_bias_
**Treatment response rate**						
Treatment						
Chemotherapy	2	0.20 (0.07, 0.54)	3.18	< 0.01	0.0%	0.458
EGFR-TKI	2	0.02 (0.01, 3.18)	1.52	0.13	80.4%	0.024
**Overall survival**						
Location						
Nuclear	9	1.95 (1.52, 2.51)	5.22	< 0.01	54.5%	0.024
Cytoplasmic	6	1.69 (0.85, 3.37)	1.50	0.13	80.3%	< 0.001
Treatment						
Chemotherapy	8	1.53 (1.08–2.17)	2.39	< 0.01	70.50%	0.001
EGFR-TKI	3	3.34 (1.06–10.52)	2.06	0.04	52.70%	0.121
N.A	4	2.51 (1.41–4.46)	3.13	< 0.01	77.30%	0.004
**PFS**						
Location						
Nuclear	5	2.15 (1.01,4.59)	4.77	0.048	88.3%	< 0.001
Cytoplasmic	2	2.82 (0.83, 9.57)	0.21	0.10	75.9%	0.042
Treatment						
Chemotherapy	3	2.00 (1.49–2.67)	4.65	< 0.01	0.00%	0.581
EGFR-TKI	2	6.90 (2.91–16.38)	4.38	< 0.01	0.00%	0.690
N.A	2	1.37 (0.43–4.36)	0.53	0.60	88.40%	0.003

Abbreviations: OR: odds ratio, HR: hazard ratio CI: confidence interval, EGFR-TKI: Epidermal growth factor receptor tyrosine kinase inhibitor. PFS: progression-free survival, N.A: the therapeutic protocol was not clearly defined

### Association between NRF2 and overall survival, progression-free survival in NSCLC

Fifteen studies were assessed for the association between NRF2 and overall survival (OS). The logistic random-effects model was applied because of significant heterogeneity in the studies (I^2^ = 67.9%, P < 0.001). The sensitivity analysis was carried out owing to the relatively large heterogeneity among the studies on OS. The pooled HR estimates were consistent without distinct fluctuation. The results indicated that a high NRF2 expression level was associated with inferior OS (HR = 1.86, 95% CI 1.44–2.41, P < 0.001) (Table 3).

Subgroup analysis based on NRF2 signal localization was also used to explore whether these potential sources of heterogeneity had an effect on overall survival. As seen in Table 4 and Fig 3B, NRF2 signal localization impacts the correlation of higher NRF2 expression with worse OS (nuclear: HR = 1.95, 95%CI 1.52–2.51, P < 0.01; cytoplasmic: HR = 1.69, 95%CI 0.85–3.37, P = 0.13). Although there was high heterogeneity within the nuclear subgroup (I^2^ = 54.5%) and cytosolic subgroup (I^2^ = 80.3%). These results indicate that the patients with a high level of nuclear NRF2 expression had a lower survival rate.

Meanwhile, we separated the studies into three subgroups according to the different treatments, including chemotherapy, EGFR-TKI, and N.A group without clearly defined treatment. The treatments impact the association of NRF2 expression with OS (chemotherapy: HR = 1.53, 95%CI 1.08–2.17, P < 0.01; EGFR-TKI: HR = 3.34, 95%CI 1.06–10.52, P = 0.04; N.A: HR = 2.51, 95%CI 1.41–4.46, P < 0.01), as shown in Table 4 and Fig 3C. Because various chemotherapies were employed in different studies and the therapeutic protocol was not clearly defined in the N.A group, the OS showed high heterogeneity among different studies. Nevertheless, the higher NRF2 expression level within these three groups of patients were inversely correlated with their OS.

Eight studies were included in the meta-analysis of progression-free survival (PFS). The random-effects model was applied because of significant heterogeneity in the studies (I^2^ = 88.1%, P < 0.001). The influence analysis was carried out showed in S2 Fig, the Haihong Yang’s study in 2011 was removed data. The heterogeneity still was high (I^2^ = 86.2%) so the logistic random-effects model was applied. The results indicated that positive NRF2 expression was associated with poor PFS (HR = 2.27, 95% CI 1.26–4.09, P = 0.006) (Table 3).

Subgroup analysis based on NRF2 signal localization and treatment was also used to explore whether these potential sources of heterogeneity had an effect on PFS. As seen in Table 4 and Fig 3D, NRF2 signal localization impacts the association of NRF2 expression with PFS (nuclear: HR = 2.15, 95% CI 1.01–4.59, P = 0.048; cytoplasmic: HR = 2.82, 95% CI 0.83–9.57, P = 0.10). The high NRF2 level in nucleus was associated with poor PFS. As shown in Fig 3E and Table 4, the treatments impact the association of NRF2 expression with PFS (chemotherapy: HR = 2.00, 95%CI 1.49–2.67, P < 0.01; EGFR-TKI: HR = 6.90, 95%CI 2.91–16.38, P < 0.01; N.A: HR = 1.37, 95%CI 0.43–4.36, P = 0.60). In the N.A group, because the therapeutic protocol was not clearly defined, the heterogeneity was high. That also caused the high heterogeneity of PFS. NRF2 higher expression was not associated with poor PFS in N.A group.

### Publication bias

Funnel plots, as well as Begg's test, was performed to evaluate potential publication bias in this meta-analysis. Most of the plots were symmetric, indicating that publication bias was low (S3 Fig). There was no evidence of significant publication bias by inspection of the formal statistical tests (Tables 3 and 4).

## Discussion

NRF2 is a transcription factor that acts as the main regulator of various antioxidant genes [38]. Several studies have shown that dysregulation of *NRF2* is closely related to human cancer [39,40]. NRF2 is involved in various tumour processes, mainly by interfering with cell proliferation and apoptosis, causing resistance to conventional chemotherapy and radiotherapy. In the case of lung cancer, clinical evidence on the relationship between NRF2 positive expression and tumour invasion or prognosis has not been thoroughly investigated.

NRF2 activators have been used in clinical trials for cancer treatment and the treatment of diseases related to oxidative stress. On the other hand, constitutive activation of NRF2 contributes to the growth of cancer cells in many types of tumours, leading to the resistance to anticancer therapy [1,41]. Considering the inconsistent reports in the literature, we conducted the meta-analysis and found that a high expression level of NRF2 was related to poor survival rate among lung cancer patients. This meta-analysis is the first systematic study to evaluate the association between NRF2 expression and clinicopathological features and overall survival in NSCLC patients. Through combined 20 publications including 2530 patients with NSCLC, our results indicate that positive NRF2 expression is correlated with high pathological metastasis, high TNM stage and increased lymph node metastasis. These findings are consistent with previous report that NRF2 has a significant impact on neoplasm invasiveness-associated features [42]. The higher expression level of NRF2 appears to be an indication of worse OS and RFS, which is consistent with the results of Wang et al. on the solid tumour [43]. The NRF2 sequences of 103 NSCLC patients was studied by Hu et al., and it was found that the NRF2 mutation rate of current and former smokers was significantly higher than that of non-smokers [44]. According to Hu, Sasaki et al., sequenced NRF2 in 262 surgically resected lung tumours confirmed that NRF2 mutations were more common in squamous cell carcinoma and smokers [45]. However, we did not detect any association between high NRF2 expression and smoking history. Since our study was entirely based on IHC data, representing the protein level of NRF2 expression, no information regarding the genetics of NRF2 can be obtained. On the other hand, only a limited number of studies were available for this meta-analysis, and such associations may become apparent with increased sample size.

There are some studies about NRF2 mutation [4,46] as a prediction for lung cancer survival. KEAP1/NRF2 mutant lung cancer is a microenvironmentally distinct, biologically heterogeneous and clinically underestimated disease that increased radioresistance in NSCLC [47–50]. The higher NRF2 expression level was significantly correlated with EGFR gene mutation in NSCLC [32]. Previous studies using microarray data to analyze the NRF2-associated genes [4,51] found that they were biomarkers for poor prognosis in NSCLC cohorts. But, the mRNA level of NRF2 alone was not correlated with the clinicopathology in NSCLC [36,52]. Thus, it is important to consider NRF2 gene mutations, mRNA level and protein level altogether in order to provide more comprehensive understanding of the prognosis among different NSCLC patients.

KEAP1/NRF2 signalling regulates glutaminolysis metabolism by inhibition of glutaminase in KRAS-KEAP1 mutant lung cancer [53] and regulates the sensitivity to EGFR-TKI. It has been reported that mutations in KEAP1/NRF2 [54] and higher expression level of DJ1 [17], NQO1 [31], TP53 [55], CUL3 [56] and PRDX5 [57] are associated with poor survival of patients with NSCLC. The synergy between the KEAP1/NRF2 and PI3K pathways drives NSCLC with an altered immune microenvironment and achieves tumour regression through suppression of immune checkpoint [58]. Patients with NSCLC usually received targeted therapy (EGFR/ALK mutation patient) or chemotherapy with cisplatin [59], with or without combined radiotherapy. The KEAP1/NRF2 mutation may define a molecular subtype that is resistant to chemotherapy [47] and therefore may rapidly develop into NSCLC. Among NSCLC patients with EGFR mutations, if KEAP1/NRF2/CUL3 co-mutation existed, the EGFR-TKI treatment showed a significantly reduced effective time window [56]. Our results also indicated that higher NRF2 expression was associated with the poor OS and PFS in both chemotherapy and EGFR-TKI treatment group. Therefore, in order to increase the sensitivity of chemotherapy and EGFR-TKI, NRF2 can be developed as a therapeutic target to benefit the NSCLC patients.

In the current study, we observed that positive NRF2 expression was associated with low treatment response rate in platinum-based chemotherapy. However, the patients underwent different chemotherapy strategies and 17 of them received surgical interventions. This suggests that chemotherapy alone may not be an effective therapy for NRF2 positive NSCLC patients [15]. The NRF2/KEAP1 pathway controls the localization of NRF2 in the nucleus and cytoplasm. The dual roles of NRF2 in tumorigenesis might therefore be caused by NRF2 shuttling between the nucleus and cytoplasm [24]. Therefore, the NRF2 location should be considered during analysis for the clinicopathological features.

There were some limitations in this meta-analysis. Firstly, in this study, the NRF2 expression was based on IHC staining data. Therefore, the choice of primary antibody and the dilution adopted could give rise to inconsistent NRF2 detection. Secondly, due to the strict selection criteria and the limited number of published studies concerning NRF2 expression and lung cancer prognosis, we were only able to include 20 published articles in this meta-analysis. Finally, the various definitions of the cut-off value of NRF2 expression in the original studies might cause additional heterogeneity and bias.

In conclusion, despite the limitations mentioned above, our meta-analysis is the first study to systematically evaluate the association between NRF2 expression and NSCLC survival. The results support an association between high expression level of NRF2 and aggressive tumor pathology in NSCLC patients. Therefore, NRF2 has the potential to become a molecular signature predicting NSCLC survival.

## Supporting information

S1 Checklist (DOC)

S1 Fig Forest plot of positive NRF2 expression and clinicopathological features. A. Forest plot of studies evaluating the relationship between NRF2 expression and gender. B. Forest plot of studies evaluating the relationship between NRF2 expression and smoking. C. Forest plot of studies evaluating the relationship between NRF2 expression and histopathology. D. Forest plot of studies evaluating the relationship between NRF2 expression and tumour differentiation type.
(TIF)

S2 Fig The influence analysis of NRF2 related studies. A., Lymph node metastasis; B., PFS
(TIF)

S3 Fig Funnel plot for publication bias test of NRF2 related studies. A., Metastasis; B., Lymph node metastasis; C., TNM stage (III~IV vs. I~II); D.,TNM stage (IV vs. III); E., Treatment response rate; F., OS; G., PFS; H., Gender; I., Smoking; J. Histopathology; K. Differentiation type.
(TIF)

S1 Table Pubmed search terms. (DOCX)

## Abbreviations

NSCLC: non-small cell lung cancer
NRF2: Nuclear factor erythroid 2-Related Factor 2
NFE2L2: Nuclear factor, erythroid 2 like 2
NQO1: NAD(P)H quinone oxidoreductase 1
HO-1: Heme oxygenase-1
GST: glutathione S-transferase
KEAP1: Kelch-like ECH-associated protein 1
PI3K: phosphatidylinositol 3-kinase
K-RAS: Kirsten retrovirus-associated DNA sequence
IHC: immunological histological chemistry
TNM stage: pathologic tumour-node-metastasis
SCC: squamous cell carcinomas
AC: adenocarcinomas
OS: overall survival
PFS: progression-free survival
HR: hazard ratio
OR: odds ratio
CI: confidence interval
EGFR-TKI: Epidermal growth factor receptor tyrosine kinase inhibitor
CR: complete response
PR: partial response
PD: progression of disease
SD: stable disease
