# Supplementary figures and images for: Prognostic and clinicopathological significance of NRF2 expression in non-small cell lung cancer: A meta-analysis
Source: PLoS One. 2020 Nov 13;15(11):e0241241. doi: 10.1371/journal.pone.0241241 (PMC7665804; doi:10.1371/journal.pone.0241241)

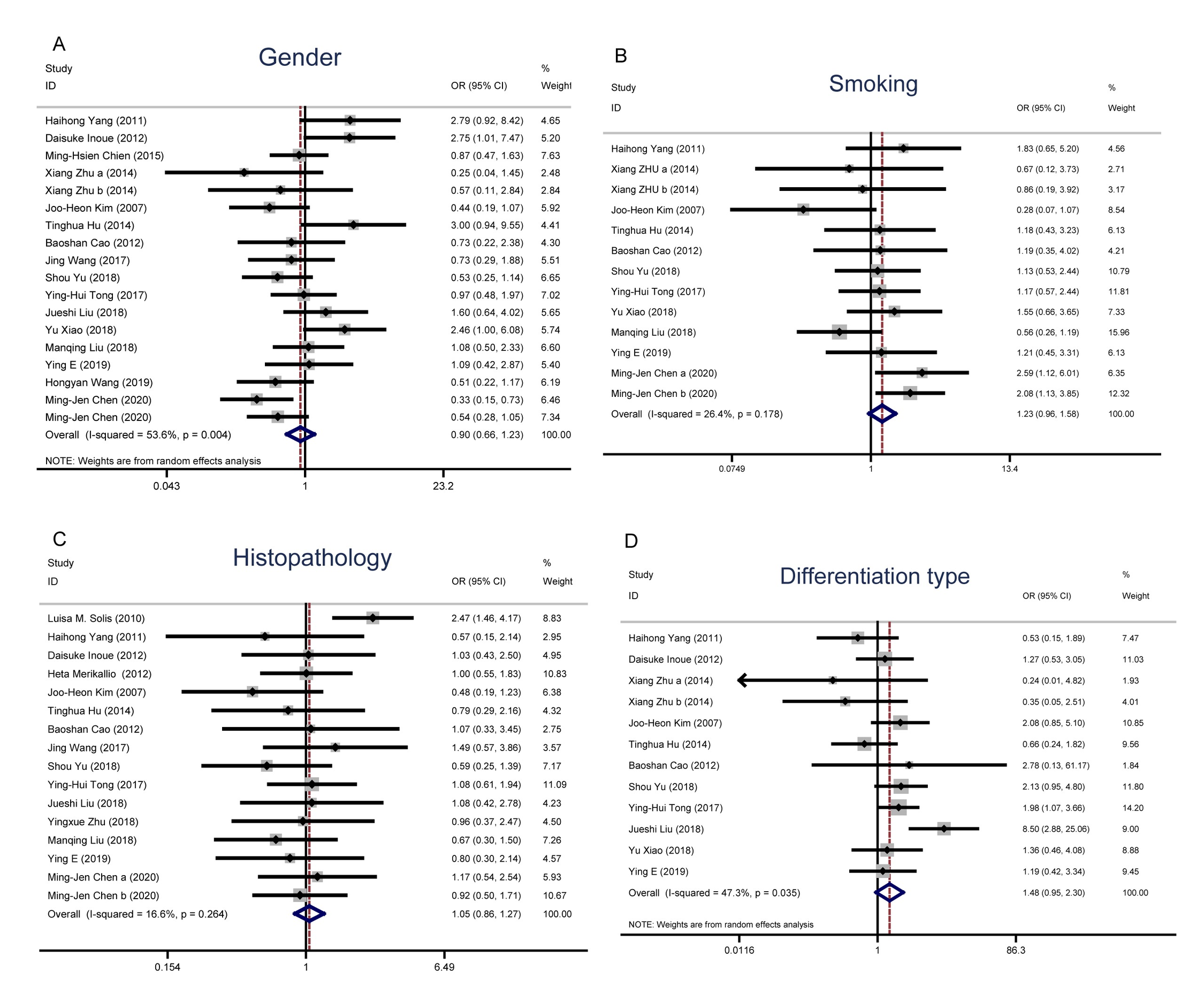

Supplement: S1 Fig — A. Forest plot of studies evaluating the relationship between NRF2 expression and gender. B. Forest plot of studies evaluating the relationship between NRF2 expression and smoking. C. Forest plot of studies evaluating the relationship between NRF2 expression and histopathology. D. Forest plot of studies evaluating the relationship between NRF2 expression and tumour differentiation type. (TIF) [file pone.0241241.s002.tif]

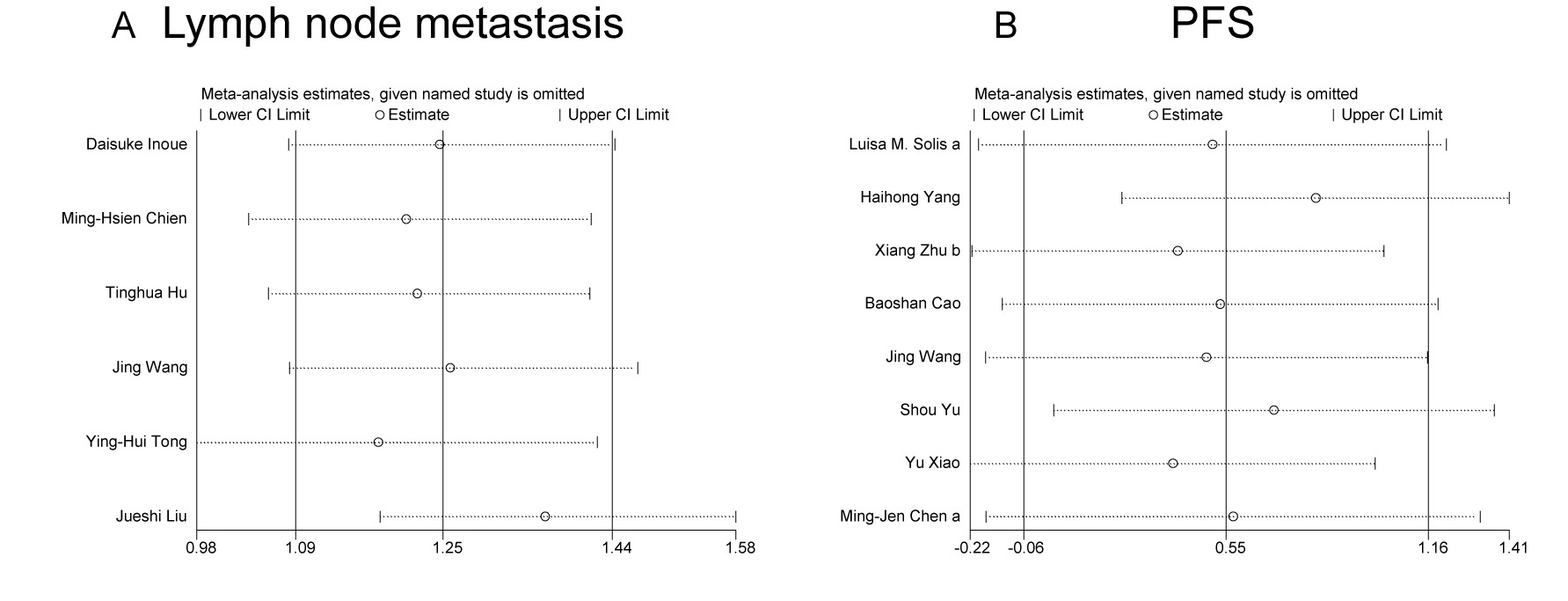

Supplement: S2 Fig — A., Lymph node metastasis; B., PFS (TIF) [file pone.0241241.s003.tif]

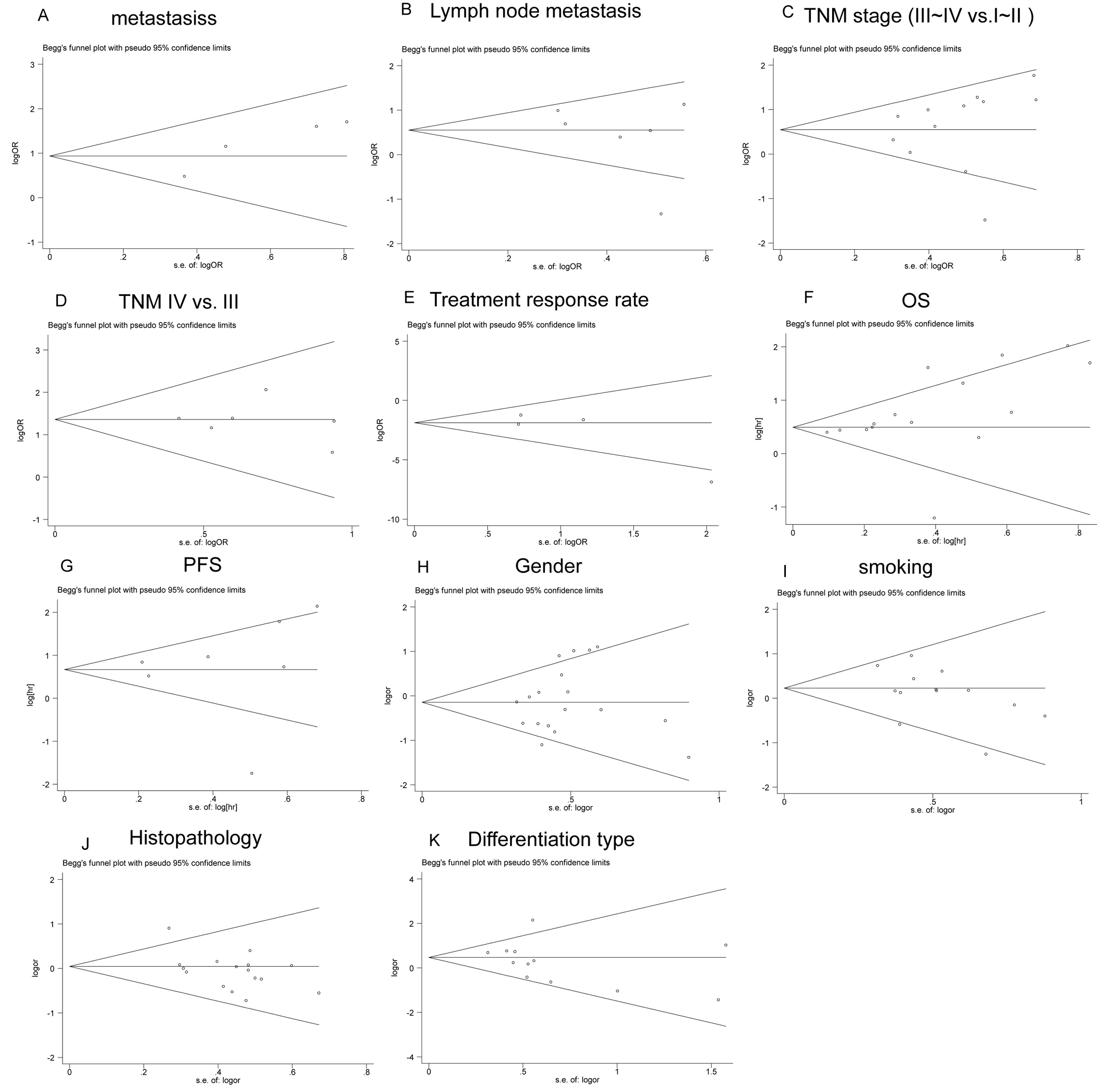

Supplement: S3 Fig — A., Metastasis; B., Lymph node metastasis; C., TNM stage (III~IV vs. I~II); D.,TNM stage (IV vs. III); E., Treatment response rate; F., OS; G., PFS; H., Gender; I., Smoking; J. Histopathology; K. Differentiation type. (TIF) [file pone.0241241.s004.tif]
