# Supplementary material for: Prognostic and clinicopathological significance of NRF2 expression in non-small cell lung cancer: A meta-analysis
Source: PLoS One. 2020 Nov 13;15(11):e0241241. doi: 10.1371/journal.pone.0241241 (PMC7665804; doi:10.1371/journal.pone.0241241)
Supplement: S1 Table — (DOCX) [file pone.0241241.s005.docx]

S1 Table. Pubmed search terms

#1 Nuclear Factor Erythroid-2-related Factor 2

#2 NFE2L2

#3 “NRF2”

#4 Search (#1 OR #2 OR #3)

#5 Lung Squamous Cell Carcinomas

#6 Lung Adenocarcinomas

#7 “Lung Cancer, Lung Carcinomas”

#8 Search (#5 OR #6)OR #7

#9 Non Small Cell Lung Carcinoma

#10 NSCLC

#11 “Lung Cancer, Lung Carcinomas”

#12 Search (#9 OR #10) OR #11

#13 Search (#8 AND #12)

#14 Search (#4 AND #13)
